# Association between dietary quality and executive functions in school-aged children with autism spectrum disorder

**DOI:** 10.3389/fnut.2022.940246

**Published:** 2022-08-04

**Authors:** Xin Wang, Xiaojing Song, Yuying Jin, Xiaoling Zhan, Muqing Cao, Xuning Guo, Siyu Liu, Xiaoxuan Ou, Tingfeng Gu, Jin Jing, Li Cai, Xiuhong Li

**Affiliations:** Department of Maternal and Child Health, School of Public Health, Sun Yat-Sen University, Guangzhou, China

**Keywords:** autism spectrum disorder, dietary quality, executive function, Diet Balance Index, children

## Abstract

**Background:**

It is well known that children with autism spectrum disorder (ASD) had executive functions deficit. However, it is still unclear whether the poor dietary quality is related to the impairment of executive functions. The current study aimed to explore the association between dietary quality and executive functions in children with ASD.

**Methods:**

A total of 106 children with ASD (7.7 ± 1.3 years) and 207 typically developing (TD) children (7.8 ± 1.3 years) were enrolled from Guangzhou, China. The Chinese version of Behavior Rating Scale of Executive function (BRIEF), the working memory subscales of the Chinese version of Wechsler Intelligence Scale for Children-Fourth Edition (WISC-IV), and the Stroop Color-Word Test (SCWT) were used to measure the participant's executive functions. The food frequency questionnaire (FFQ) was used to collect the dietary intake information, and the Chinese Diet Balance Index (DBI_16) was used to evaluate the dietary quality. Generalized linear models were used to estimate the association between dietary quality and executive functions.

**Results:**

In children with ASD, Low Bound Score (LBS) was positively correlated with the working memory subscale score of BRIEF (β = 0.23, 95% CI: 0.02–0.44, *P* < 0.05), while High Bound Score (HBS) and LBS were positively correlated with the organizable subscale score of BRIEF (β = 0.44, 95% CI: 0.11–0.77, *P* < 0.01; β = 0.19, 95% CI: 0.01–0.37, *P* < 0.05). Compared to TD children, children with ASD had a higher proportion of moderate and high levels of insufficient dietary intake (moderate level, 37.7% vs. 23.2%, high level, 4.7% vs. 1.4%) and moderate level of unbalanced dietary intake (36.8% vs.21.3%), higher scores on all subscales of BRIEF (*P* < 0.01), and lower score on the working memory (81.3 ± 32.3 vs. 104.6 ± 12.5, *P* < 0.01), while there was no difference on the SCWT.

**Conclusion:**

Poor dietary quality was associated with the impairment of working memory and organizational capacity in children with ASD. This study emphasized the importance of dietary quality in executive functions among children with ASD, and attention should be paid to improving their dietary quality.

## Introduction

Autism spectrum disorder (ASD) is a developmental disorder characterized by social communication deficits and repetitive/unusual sensory-motor behaviors (1), with a median prevalence of 1% (2). ASD is highly disabling, resulting in a heavy public health burden (3). Numerous studies have shown that children with ASD had abnormal executive functions. Executive functions refer to a set of cognitive abilities responsible for goal-directed behavior (e.g., working memory, attention, planning, response inhibition, mental flexibility, and self-monitoring) (4, 5). Executive functions have an important role in everyday behaviors across the life span (6). Literature showed that the impairment of executive functions might associate with worse core symptoms (7, 8), poor quality of life (9), and impaired mental and physical health (10–12) in children with ASD. Thus, exploring the risk factors for the impairment of executive functions in children with ASD is become increasingly important and will help to propose feasible treatment intervention plans for children with ASD in the future.

Although there were many studies on dietary problems in children with ASD, the traditional dietary evaluation was based on specific foods or nutrient intake, with inconsistent results. Evans et al. (13) compared dietary patterns among American children and found that children with ASD consumed significantly more sweetened beverages and snack foods and fewer fruits and vegetables than typically developing (TD) children. Meguid et al. (14) found that Egyptian children with ASD consumed more carbohydrates but lower protein than healthy controls. Tsujiguchi et al. (15) found that children with ASD consumed more carbohydrates but slightly less protein, fat, minerals, and vitamins than children without ASD in Japan. A nutritional survey in Chongqing, China suggested that Children with ASD consumed fewer macronutrients and vitamin A (16). Because of dietary quality's support on the morphological development, neurochemistry, and neurophysiology of the human brain (17), effects on synaptic plasticity (18), influence on the gut microbiome (19), it was suggested that dietary quality might be associated with executive functions (20).

Up to now, there are many studies exploring the relationship between dietary quality and executive functions in TD children (see Supplementary Table S1). Riggs et al. (21) found that the overall executive function measured by Behavior Rating Scale of Executive function (BRIEF) was negatively correlated with the intake of high-calorie snacks, and positively correlated with the intake of fruits and vegetables. Guerrieri et al. (22) measured inhibitory control using the stop-signal task and found that there was no relationship between inhibition and calorie intake. However, another longitudinal study showed that there was a positive relationship between inhibitory control measured by BRIEF and the intake of “high-calorie, low nutrient snacks” (23). Such inconsistent conclusions also exist in working memory, cognitive flexibility, and other components of executive functions.

Notably, comparatively little is known about the association between dietary quality and executive functions among children with ASD. Firstly, few studies have focused on children with ASD. Secondly, the dietary assessment methods used in previous studies cannot accurately reflect the complexity of dietary quality. Thirdly, there was a lack of overall assessment of the executive functions in children. To address these gaps, we used the revised Chinese Diet Balance Index (DBI_16) to comprehensively evaluate the dietary quality of Children with ASD, including indices of insufficient, excessive, or imbalanced dietary intake. We also used the parent-reported questionnaire of BRIEF and face-to-face experimental tasks to comprehensive evaluate the executive functions. BRIEF included 2 indices: the Behavioral Regulation Index (BRI) and the Metacognition Index (MI). BRI reflects the ability of the child to shift cognitive set and modulate emotions and behaviors *via* appropriate inhibitory control. MI indicates the child's ability to initiate, plan, organize, and sustain future-oriented problem-solving in working memory. Corresponding to the two indices, the face-to-face experimental tasks used in the current study included working memory subscales of the Chinese version of Wechsler Intelligence Scale for Children-Fourth Edition (WISC-IV) and the Stroop Color-Word Test (SCWT), a classic inhibitory control task.

In short, the purpose of the study was to explore the relationship between dietary quality and executive function in children with ASD.

## Methods

### Participants

We used the baseline data of the ongoing cohort of “the Guangzhou Study of Children with ASD” in Guangzhou, China. We selected a subsample of 107 children with ASD and 209 TD children from Jan, 2021 to Sep, 2021. One child with ASD missed data from subscales of BRIEF and 2 TD children with Intelligence Quotient (IQ) below 80 were excluded. Finally, 106 children with ASD (89 boys and 17 girls) and 207 TD children (113 boys and 94 girls) were included in the final analysis. All the 313 participants took part in SCWT. But only 76 children with ASD and 173 TD children finished SCWT, because it was based on literacy. We obtained working memory index and a full-scale intelligence quotient (FSIQ) of 99 children with ASD and 207 TD children, who completed WISC-IV. All the children with ASD were diagnosed by a combination of the Childhood Autism Rating Scale (CARS) and an expert clinician. Their diagnosis was further confirmed by two professional child psychiatrists using the Diagnostic and Statistical Manual of Mental Disorders, Fifth Revision (DSM-5) criteria. Detailed inclusion criteria for both groups were as follows: (1) chronological age between 6 years 0 months and 12 years 0 months; (2) voluntary participation of the children's parents. Exclusion criteria included: (1) dyslexia, seizures, head trauma, cerebral palsy, severe visual or auditory impairment, other movement disorders; (2) genetic or chromosomal abnormalities.

### Procedure

Children underwent face-to-face measures performed by trained psychometrists or research assistants at the research center. Information on demographic, physical activity, executive functions, and dietary intake status was obtained through in-person interviews with primary caregivers or validated tools/questionnaires. All the parents of the participants signed informed consent forms. The study was approved by the Ethical Review Committee for Biomedical Research, Sun Yat-Sen University (2015-No.29).

### Measures

#### Assessment of executive functions

We assessed executive functions by parent-reported questionnaire and face-to-face experimental tasks. The rating scale we used was the Chinese version of BRIEF for children aged 6 to 18 years. The BRIEF is a validated 86-item questionnaire with the Cronbach α coefficient ranging from 0.74 to 0.96 (24). It includes the following subscales: inhibit, shift, emotional control, initiate, working memory, plan/organize, organization of materials, and monitor. The subscales form 2 indices: BRI and MI. The Global Executive Composite (GEC) combines the 2 indices and represents the summary measure of executive functions. The 86 items are rated using the following responses: never, sometimes, or often, coded 1, 2, or 3, respectively. A higher score indicates greater perceived impairment of executive functions. The subscales and total scores were calculated and standardized into Z-scores.

Children's executive functions were also assessed by face-to-face experimental tasks including working memory subscales of the Chinese version of WISC-IV, and the classical inhibitory control tasks (i.e., SCWT). The WISC-IV's working memory index is a commonly index to test working memory of executive functions. Working memory subscales include Digit Span and Letter-Number Sequencing subtests; Digit Span assesses short-term working memory by asking the child to repeat a series of increasingly long number sequences forward and then backward; Letter-Number Sequencing is a well-validated measure of manipulation working memory. The tester read a series of numbers and letters, then asked the child to recall the numbers in ascending order and the letters in alphabetical order. The SCWT is a classical test of inhibition (25), which was computerized using E-Prime 2.0 in current study. This test consisted of three subtasks, namely reading the color words, naming the colors, and naming the color of the ink rather than the words. Each subtask consisted of 10 trials, and each trial presented a color or a color word. The interviewer manipulated buttons on the keyboard to record responses and asked the participants to respond as accurately and quickly as possible. We calculated the differences between the incongruent and neutral conditions in terms of mean reaction time (I-NRT) and correct rate (N-I %Cor).

#### Assessment of dietary quality

The dietary intake information of children was collected by a parent-reported Food Frequency Questionnaire (FFQ). The FFQ contains 20 kinds of food such as cereal, vegetables, fruit, animal food, condiments. The questionnaire had been validated before the study (26), the average Intraclass Correlation Coefficient (ICC) for test-retest reliability was 0.398. The respondent of FFQ (i.e., the participant's parent or primary caretaker) needed to indicate how many times last seven days the participant ate a given food and to describe the size of the usual serving relative to a standard serving on the days they had eaten the food (1 serving = 1 egg size). Then we calculated the daily intake of various foods [intake frequency (times/week) × average intake per time /7].

We assessed dietary quality by the DBI_16 revised by He et al. (27), which consisted of eight food group-level indicators including cereals, vegetable and fruit, dairy and soybean, animal food, empty energy food (cooking oil, alcoholic beverage), condiments (addible sugar, salt), variety and water. The DBI_16 indicators were calculated by the insufficient, appropriate and excessive dietary intake based on the recommendations of Dietary Guidelines for Chinese Residents-2016. Being evaluated to insufficient, excessive or imbalanced dietary intake according to the recommendations is considered to be poor dietary quality. For foods that are emphasized as “appropriate amount” in the Dietary Guidelines, the index value should reflect both insufficient intake and excessive intake, so there are positive and negative bidirectional values, such as cereals and animal foods (including red meat and products, poultry and game, fish and shrimp, and eggs); For foods that are emphasized as “more to eat” in the Dietary Guidelines, the index value mainly evaluates the degree of insufficient, so the index value is negative, such as vegetable and fruit, dairy and soybean, variety and water; The index values are positive for foods that are highlighted as “less to eat” in the Dietary Guidelines, such as cooking oil and salt, because they focus on assessing the degree of excess. According to the DBI_16 standard, the dietary intake of children with ASD and TD children was calculated to obtain the scores of each single index of DBI_16 and combine to values of the high bound score (HBS), low bound score (LBS), and diet quality distance (DQD). HBS is the positive-sum value of all the DBI_16 index scores, reflecting the situation of children's excessive dietary intake, with the score ranging from 0 to 44. LBS is the absolute value of the sum of all negative values in the DBI_16 index score, which reflects the insufficient dietary intake of children, with the score ranging from 0 to 72. DQD is the absolute value of all the DBI_16 index scores, which comprehensively reflects the overall situation of children's dietary intake, and the score ranges from 0 to 96. And HBS, LBS, and DQD were divided into five levels: no problem (= 0), almost no problem (HBS: 1~9, LBS: 1~14, DQD: 1~19), low level (HBS: 10~18, LBS: 15~29, DQD: 20~38), moderate level (HBS: 19~27, LBS: 30~43, DQD: 39~57) and high level (HBS: 28~44, LBS: 44~72, DQD: 58~96). (The detail of DBI_16 was shown in Supplementary Table S2).

#### Assessment of covariates

##### Demographic information

The children's age, gender, maternal age and education level, paternal age and education level, and per capita monthly household income were reported by parents *via* questionnaire.

##### Anthropometric measurements

Anthropometric measurements were taken by trained staff according to the standard procedure of anthropometric procedures and data collection developed by WHO. The weight and height were averaged by two measurements, and the body mass index (BMI) was estimated by dividing weight (kg) by height^2^ (m^2^). The definitions of overweight and obesity were using the screening thresholds for overweight and obesity in Chinese school-age children and adolescents released by the National Health and Family Planning Commission in 2018 (28).

##### Physical activity

Physical activity was assessed by the International Physical Activity Questionnaire short format which included vigorous physical activity, moderate physical activity, and walking. For the above activities, respondents were asked to review the activities of at least 10 min each time in the past 7 days and answer the number of days of activities and the average daily duration. Based on the total metabolic equivalent (MET), physical activity status was classified into three categories: low, moderate, and high (29).

##### Intelligence quotient

IQ was assessed *via* the Chinese Version of WISC-IV, which provides a full-scale intelligence quotient (FSIQ) based on the sum of scores from the 10 core subtests (30). FSIQ < 70 was defined as intellectual disorder (ID).

### Statistical analyses

To compare the differences in characteristics between ASD and TD children, we calculated means and standard deviations for continuous variables using independent sample *t*-tests, and calculated percentages for categorical variables using chi-square tests. We used generalized linear models to compare the differences in dietary quality and executive functions between the two groups and to investigate the associations between dietary quality and executive functions in both groups. We fitted the crude model without any adjustments and adjusted the model by adjusting for child's age, sex, maternal education level, paternal education level, family income, category of physical activity, BMI, and with or without ID (FSIQ ≥ 70 / <70). The results were presented as coefficient estimates (β) with a 95% confidence interval (CI). For all analyses, the statistical significance was set at *P*-value < 0.05 (two-sided). We conducted all statistical analyses with R 4.0.3 statistical software (31).

## Results

### Demographic characteristics

The demographic characteristics of the included children are shown in Table 1. The mean age of children with ASD was 7.7 ± 1.3 years. There were 84.0% of children with ASD were boys. Compared with TD children, children with ASD had lower scores in the FSIQ (92.0 ± 18.8 vs. 112.9 ± 12.8, *P* < 0.01), a higher proportion of the low and moderate levels of physical activity (10.4% vs. 3.4% and 43.4% vs. 36.2%, *P* < 0.01), and lower maternal education level (41.5% vs. 24.6%, *P* < 0.01) and family economic situation (57.5% vs. 25.1%, *P* < 0.01). There were no differences between groups in children's age, category of BMI, or paternal education level.

**Table 1:** Demographic characteristics of children with ASD and TD children.

**Characteristics**	**ASD**	**TD**	***P* value**
	**Means (SD)/*N* (%)**	**Means (SD)/*N* (%)**	
	**N = 106**	**N = 207**	
Age	7.7 (1.3)	7.8 (1.3)	0.86
Sex			
Boy	89 (84.0)	113 (54.6)	**< 0.01**
Girl	17 (16.0)	94 (45.4)	
FSIQ	92.0 (18.8)	112.9 (12.8)	**< 0.01**
BMI			0.07
Underweight	10 (9.4)	26 (12.7)	
Normal	68 (64.2)	146 (71.6)	
Overweight	12 (11.3)	19 (9.3)	
Obesity	16 (15.1)	13 (6.4)	
Level of physical activity			**< 0.01**
Low	11 (10.4)	7 (3.4)	
Moderate	46 (43.4)	75 (36.2)	
High	49 (46.2)	125 (60.4)	
Maternal education level			**< 0.01**
Below bachelor	44 (41.5)	51 (24.6)	
Bachelor degree or above	62 (58.5)	156 (75.4)	
Paternal education level			
Below bachelor	40 (37.7)	73 (35.4)	0.69
Bachelor degree or above	66 (62.3)	133 (64.6)	
Per capita monthly household income			**< 0.01**
≤ ¥8,000 /month	61 (57.5)	52 (25.1)	
> ¥8,000 /month	45 (42.5)	155 (74.9)	

ASD, autism spectrum disorder; TD, typically developing; SD, standard deviation; FSIQ, full scale intelligence quotient; BMI, body mass index.

The bold values represented statistically significant difference between children with ASD and TD children.

### The association of DBI_16 indicators with executive functions in children with ASD and TD children

Table 2 shows the association between DBI_16 indicators and executive functions in children with ASD. LBS was positively associated with the working memory subscale score of BRIEF (β = 0.23, 95% CI: 0.02–0.44, *P* < 0.05). HBS and LBS had positive associations with the organizable subscale score of BRIEF (β = 0.44, 95% CI: 0.11–0.77, *P* < 0.01; β = 0.19, 95% CI: 0.01–0.37, *P* < 0.05) in the adjusted model, while other indicators had no association with other components of executive functions.

**Table 2:** The association of DBI_16 indicators with executive functions in children with ASD.

**Executive functions**	**HBS**		**LBS**		**DQD**	
	**Crude model**	**Adjust model**	**Crude model**	**Adjust model**	**Crude model**	**Adjust model**
	**β (95% CI)**	**β (95% CI)**	**β (95% CI)**	**β (95% CI)**	**β (95% CI)**	**β (95% CI)**
**BRIEF**						
GEC	0.15	0.24	0.07	0.10	−0.01	−0.02
	(−0.20, 0.51)	(−0.14, 0.62)	(−0.13, 0.26)	(−0.11, 0.31)	(−0.21, 0.20)	(−0.23, 0.18)
BRI	−0.02	0.01	−0.08	−0.09	0.09	0.08
	(−0.43, 0.39)	(−0.42, 0.45)	(−0.30, 0.15)	(−0.32, 0.15)	(−0.15, 0.33)	(−0.16, 0.31)
Inhibit	0.06	0.13	0.08	0.09	−0.05	−0.07
	(−0.41, 0.54)	(−0.39, 0.65)	(−0.18, 0.34)	(−0.19, 0.37)	(−0.32, 0.23)	(−0.35, 0.20)
Shift	−0.20	−0.19	−0.22	−0.21	0.15	0.18
	(−0.61, 0.21)	(−0.62, 0.24)	(−0.44, 0.00)	(−0.44, 0.03)	(−0.09, 0.38)	(−0.06, 0.41)
Emotion control	0.04	0.05	−0.22	−0.13	0.13	0.11
	(−0.37, 0.45)	(−0.39, 0.48)	(−0.44, 0.00)	(−0.37, 0.10)	(−0.10, 0.37)	(−0.12, 0.35)
MI	0.24	0.36	−0.09	0.20	−0.06	−0.08
	(−0.11, 0.59)	(−0.02, 0.73)	(−0.31, 0.14)	(−0.00, 0.40)	(−0.27, 0.14)	(−0.28, 0.12)
Initiate	−0.01	−0.01	0.06	0.13	−0.08	−0.07
	(−0.41, 0.39)	(−0.45, 0.43)	(−0.16, 0.28)	(−0.11, 0.36)	(−0.32, 0.15)	(−0.30, 0.16)
Working memory	0.11	0.28	0.16	**0.23**	−0.13	−0.15
	(−0.28, 0.50)	(−0.11, 0.68)	(−0.05, 0.38)	(0.02, 0.44)^*^	(−0.36, 0.09)	(−0.37, 0.08)
Plan/organize	0.24	0.36	0.10	0.15	0.00	−0.02
	(−0.15, 0.63)	(−0.06, 0.79)	(−0.12, 0.31)	(−0.08, 0.38)	(−0.23, 0.23)	(−0.25, 0.20)
Organize	**0.44**	**0.44**	**0.21**	**0.19**	−0.04	−0.09
	(0.14, 0.74)^***^	(0.11, 0.77)^**^	(0.04, 0.38)^*^	(0.01, 0.37)^*^	(−0.22, 0.14)	(−0.27, 0.09)
Monitor	0.26	0.40	0.11	0.19	−0.03	−0.04
	(−0.14, 0.65)	(−0.03, 0.83)	(−0.10, 0.33)	(−0.08, 0.39)	(−0.26, 0.20)	(−0.27, 0.19)
**SCWT^a^**						
N–I %Cor	−0.00	−0.00	0.00	0.00	−0.00	−0.00
	(−0.01, 0.00)	(−0.01, 0.00)	(−0.00, 0.00)	(−0.00, 0.00)	(−0.00, 0.00)	(−0.00, 0.00)
I–NRT	3.73	−0.40	−2.38	−5.25	3.39	6.14
	(−16.53, 23.99)	(−23.52, 22.72)	(−13.28, 8.51)	(−16.95, 6.45)	(−8.57, 15.35)	(−6.63, 18.91)
Working memory test^b^	0.80	−0.09	0.44	0.06	−0.08	−0.09
	(−0.46, 2.07)	(−1.07, 0.89)	(−0.25, 1.13)	(−0.47, 0.59)	(−0.81, 0.65)	(−0.64, 0.46)

DBI_16, The Chinese Dietary Balance Index_16; LBS, Low Bound Score; HBS, High Bound Score; DQD, Diet Quality Distance; CI, confidence interval; GEC, Global executive component; BRI, Behavioral regulation index; MI, Metacognition index; SCWT, Stroop Color-Word test; I-NRT, the difference between the incongruent and neutral conditions in terms of mean reaction time; N-I %Cor, the difference between the incongruent and neutral conditions in terms of correct rate.

Crude model: without adjustment.

Adjust model: adjusted for child's age, sex; maternal education level, paternal education level, family income, level of physical activity, BMI category and with or without intellectual disorder (FSIQ≥ 70/ <70).

* P < 0.05,

** P < 0.01,

*** P < 0.001.

a 76 autistic children finished SCWT;

b 99 autistic children finished Working memory test.

The bold values represented statistically significant association between executive functions and the DBI_16 indicators.

In addition, there was a null association between DBI_16 indicators and all executive function scores in the adjusted models in TD children (see Table 3).

**Table 3:** The association of DBI_16 indicators with executive function in TD children.

**Executive functions**	**HBS**		**LBS**		**DQD**	
	**Crude model**	**Adjust model**	**Crude model**	**Adjust model**	**Crude model**	**Adjust model**
	**β (95% CI)**	**β (95% CI)**	**β (95% CI)**	**β (95% CI)**	**β (95% CI)**	**β (95% CI)**
**BRIEF**						
GEC	0.04	0.09	−0.12	−0.08	0.14	0.12
	(−0.21, 0.30)	(−0.17, 0.36)	(−0.27, 0.03)	(−0.23, 0.07)	(−0.01, 0.28)	(−0.04, 0.27)
BRI	−0.07	−0.02	−0.09	−0.06	0.06	0.05
	(−0.30, 0.17)	(−0.26, 0.22)	(−0.22, 0.05)	(−0.19, 0.08)	(−0.07, 0.20)	(−0.09, 0.19)
Inhibit	0.09	0.11	−0.06	−0.04	0.09	0.07
	(−0.18, 0.35)	(−0.16, 0.38)	(−0.22, 0.09)	(−0.19, 0.12)	(−0.06, 0.25)	(−0.08, 0.23)
Shift	−0.19	−0.18	−0.07	−0.03	0.01	−0.02
	(−0.43, 0.05)	(−0.43, 0.08)	(−0.21, 0.07)	(−0.18, 0.11)	(−0.13, 0.15)	(−0.17, 0.12)
Emotion control	−0.12	−0.05	−0.08	−0.07	0.04	0.05
	(−0.37, 0.12)	(−0.31, 0.20)	(−0.22, 0.06)	(−0.21, 0.08)	(−0.11, 0.18)	(−0.10, 0.20)
MI	0.13	0.17	−0.13	−0.09	**0.18**	0.15
	(−0.15, 0.40)	(−0.11, 0.46)	(−0.29, 0.03)	(−0.25, 0.07)	(0.02, 0.34)^*^	(−0.01, 0.32)
Initiate	0.08	0.08	−0.14	−0.10	**0.16**	0.14
	(−0.20, 0.36)	(−0.22, 0.38)	(−0.30, 0.02)	(−0.27, 0.07)	(0.00, 0.32)^*^	(−0.04, 0.21)
Working memory	0.08	0.14	**−0.16**	−0.11	**0.19**	0.16
	(−0.20, 0.36)	(−0.15, 0.42)	**(−0.32**, −0.00)^***^	(−0.27, 0.05)	(0.03, 0.35)^*^	(−0.01, 0.32)
Plan/organize	0.13	0.20	−0.10	−0.06	0.14	0.13
	(−0.17, 0.43)	(−0.11, 0.51)	(−0.27, 0.08)	(−0.24, 0.12)	(−0.03, 0.31)	(−0.05, 0.31)
Organize	0.12	0.11	−0.09	−0.08	0.13	0.12
	(−0.14, 0.39)	(−0.17, 0.40)	(−0.26, 0.08)	(−0.24, 0.08)	(−0.03, 0.28)	(−0.04, 0.29)
Monitor	0.08	0.15	−0.09	−0.04	0.12	0.10
	(−0.21, 0.38)	(−0.15, 0.45)	(−0.26, 0.08)	(−0.22, 0.13)	(−0.05, 0.29)	(−0.08, 0.27)
**SCWT^a^**						
N–I %Cor	0.00	0.00	0.00	−0.00	0.00	0.00
	(−0.00, 0.00)	(−0.00, 0.00)	(−0.00, 0.00)	(−0.00, 0.00)	(−0.00, 0.00)	(−0.00, 0.00)
I–NRT	−2.75	−0.10	1.16	1.48	−2.17	−1.56
	(−15.03, 9.52)	(−13.20, 12.99)	(−6.30, 8.61)	(−6.31, 9.28)	(−9.61, 5.28)	(−9.45, 6.33)
Working memory test^b^	0.15	−0.08	0.13	−0.08	−0.08	0.05
	(−0.22, 0.52)	(−0.36, 0.19)	(−0.09, 0.34)	(−0.24, 0.08)	(−0.29, 0.14)	(−0.11, 0.22)

DBI_16, The Chinese Dietary Balance Index_16; LBS, Low Bound Score; HBS, High Bound Score; DQD, Diet Quality Distance; CI, confidence interval; GEC, Global executive component; BRI, Behavioral regulation index; MI, Metacognition index; SCWT, Stroop Color-Word test; I-NRT, the difference between the incongruent and neutral conditions in terms of mean reaction time; N-I %Cor, the difference between the incongruent and neutral conditions in terms of correct rate.

Crude model: without adjustment; Adjust model: adjusted for child's age, sex; maternal education level, paternal education level, family income, level of physical activity, BMI category and FSIQ.

* P < 0.05,

*** P < 0.001.

a 173 TD children finished SCWT;

b 207 TD children finished Working memory test.

The bold values represented statistically significant association between executive functions and the DBI_16 indicators.

### The comparison of DBI_16 indicators and components between children with ASD and TD children

**Figure 1:**
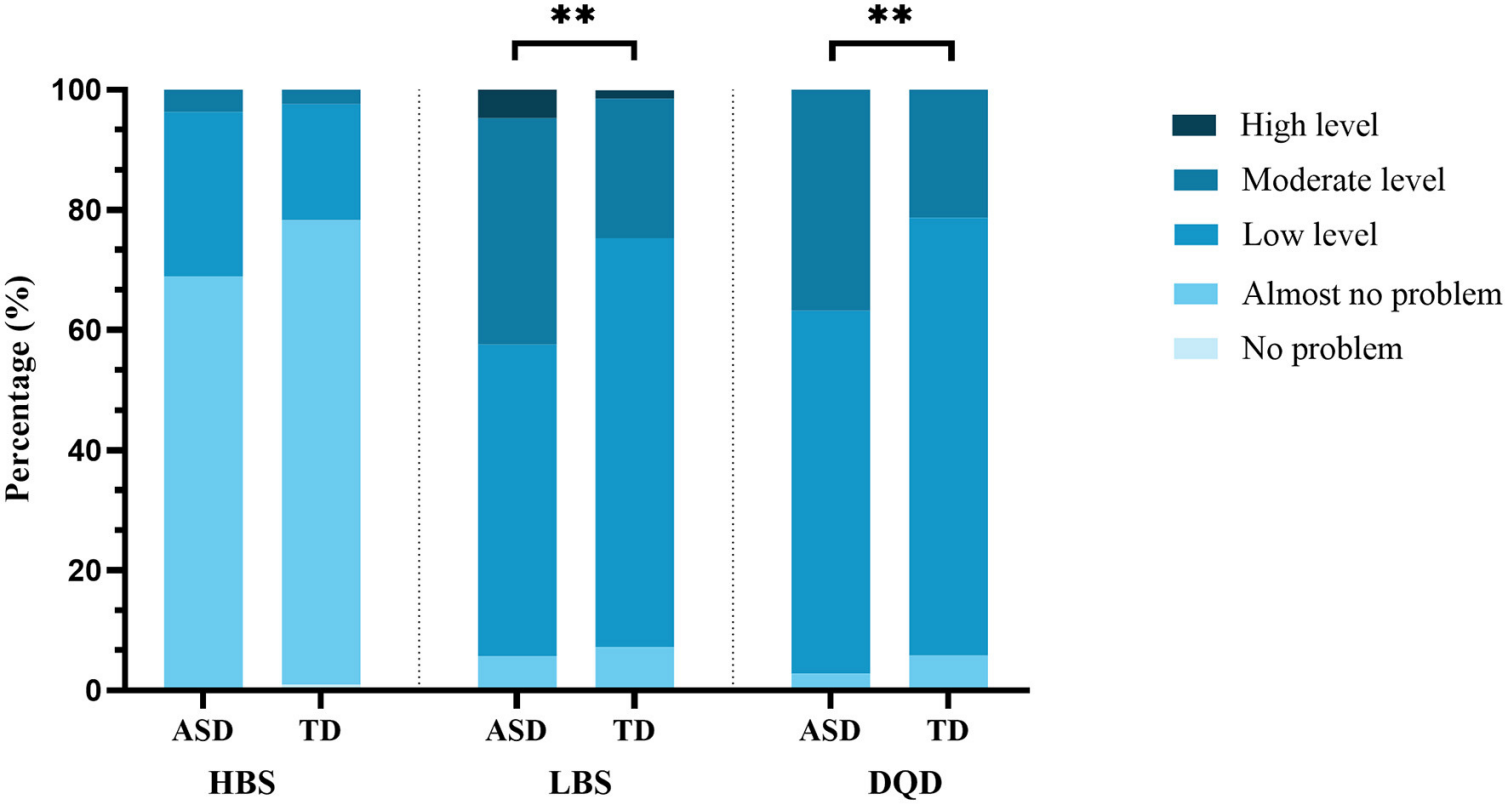
Comparison of DBI_16 indicators among children with ASD and TD children. ASD, autism spectrum disorder; TD, typically developing; LBS, Low Bound Score; HBS, High Bound Score; DQD, Diet Quality Distance. **P < 0.01.

**Figure 2:**
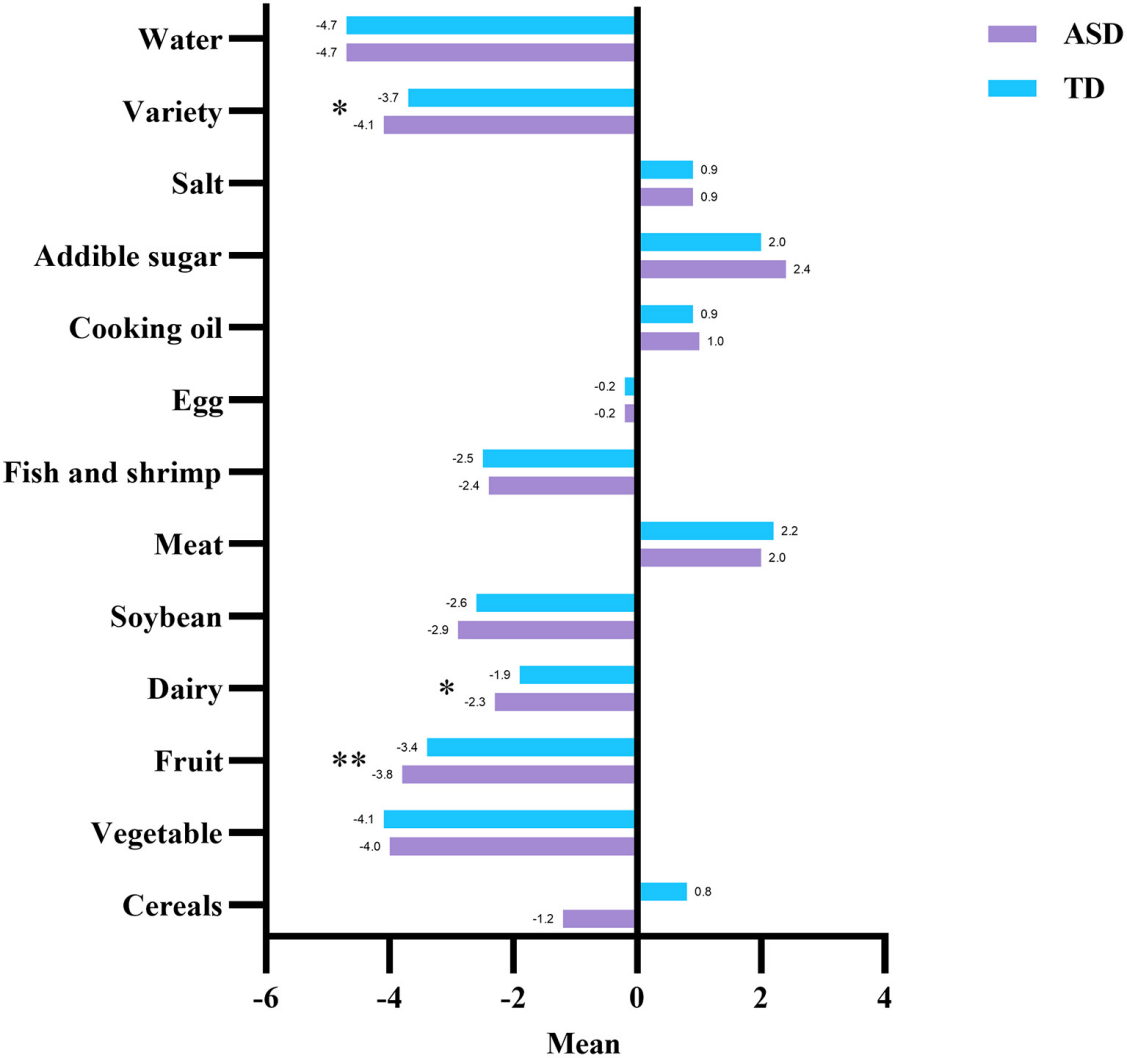
Comparison of DBI_16 components among children with ASD and TD children. ASD, autism spectrum disorder; TD, typically developing; LBS, Low Bound Score; HBS, High Bound Score; DQD, Diet Quality Distance. *P < 0.05; **P < 0.01.

As shown in Figure 1 (detailed data are shown in Supplementary Table S3), the distribution of LBS and DQD had a significant difference between children with ASD and TD children. Children with ASD had a higher proportion of the moderate and high levels of insufficient dietary intake (moderate level, 37.7% vs. 23.2%, high level, 4.7% vs. 1.4%) and the moderate level of unbalanced dietary intake (36.8% vs.21.3%) comparing with TD children. As shown in Figure 2 (detailed data are shown in Supplementary Table S4), children with ASD had more insufficient dietary intake in the fruit (−3.8 ± 1.4 vs. −3.4 ± 1.5, *P* < 0.01), dairy (−2.3 ± 1.8 vs. −1.9 ± 1.6, *P* < 0.05) and dietary variety (−4.1 ± 1.9 vs. −3.7 ± 1.9, *P* < 0.05) categories than TD children.

### The comparison of executive functions between children with ASD and TD children

Table 4 shows the comparison of executive functions in children with ASD and TD children. Compared to TD children, children with ASD had higher BRIEF total score (66.3 ± 9.2 vs. 53.9 ± 8.6, *P* < 0.01), dimensions scores (BRI, 60.8 ± 10.5 vs. 48.6 ± 7.8, MI, 67.9 ± 9.1 vs. 56.8 ± 9.3, *P* < 0.01) and all subscale scores, lower working memory score (81.3 ± 32.3 vs. 104.6 ± 12.5, *P* < 0.01). There was no difference in reaction time and correct rate of SCWT between the two groups.

**Table 4:** The comparison of executive function in children with ASD and TD children.

**Executive function**	**ASD/Mean** **(SD)**	**TD/Mean** **(SD)**	***P* value**
	**(*N* = 106)**	**(*N* = 207)**	
**BRIEF**			
GEC	66.3 (9.2)	53.9 (8.6)	**< 0.01**
BRI	60.8 (10.5)	48.6 (7.8)	**< 0.01**
Inhibit	66.0 (12.3)	50.2 (9.0)	**< 0.01**
Shift	57.6 (10.5)	48.9 (8.2)	**< 0.01**
Emotion control	54.8 (10.6)	47.5 (8.3)	**< 0.01**
MI	67.9 (9.1)	56.8 (9.3)	**< 0.01**
Initiate	61.4 (10.4)	52.6 (9.4)	**< 0.01**
Working memory	67.9 (10.1)	54.4 (9.4)	**< 0.01**
Plan/Organize	68.7 (10.2)	58.9 (10.1)	**< 0.01**
Organization	57.1 (8.0)	54.1 (9.0)	**< 0.01**
Monitor	71.1 (10.2)	58.8 (9.9)	**< 0.01**
**SCWT^a^**			
I-NRT/ ms	401.1 (464.3)	407.9 (384.1)	0.91
N-I %Cor	0.9 (0.1)	0.9 (0.1)	0.09
Working memory test^b^	81.3 (32.3)	104.6 (12.5)	**< 0.01**

SD, Standard deviation; GEC, Global executive component; BRI, Behavioral regulation index; MI, Metacognition index; SCWT, Stroop Color-Word test; I-NRT, the difference between the incongruent and neutral conditions in terms of mean reaction time; N-I %Cor, the difference between the incongruent and neutral conditions in terms of correct rate.

a 76 autistic children and 173 TD children finished SCWT;

b 99 autistic children and 207 TD children finished Working memory test.

The bold values represented statistically significant difference between children with ASD and TD children.

## Discussion

The current study showed that higher HBS was associated with worse organizable ability, while higher LBS was associated with worse organizable ability and working memory in children with ASD. Children with ASD had more problems of insufficient and unbalanced dietary intake compared to TD children. Children with ASD also had lower scores of BRIEF and working memory than TD children, while there was no significant difference in SCWT. To our knowledge, it was the first study using the DBI_16 scores to evaluate the dietary quality of Chinese children with ASD and explore the association of dietary quality with executive functions in children with ASD.

The most important finding of this study was that excessive and insufficient dietary intake in children with ASD was both positively correlated with impairment of organizable ability and working memory. These two components of executive function were all belong to Metacognition Index, which indicated that diet quality might have more effect on the ability of initiate, plan, organize, and sustain future-oriented problem-solving in working memory. The possible reason was that MI is particularly sensitive to brain-based deficits and disruptions in brain connectivity (32), and poor diet quality might associate with impairment of brain function (17). In future, more studies are needed to explore the underlying mechanisms involved. It is worth noting that the associations between dietary quality and executive functions were only observed in BRIEF, not in working memory test of WISC-IV. A possible reason is that BRIEF has higher ecological effect compared to psychometric tests (33), it is based on more representative environmental situations (34). But as an objectively psychometric tests, working memory test of WISC-IV may better reflect the state of brain development (33). Consistent with previous animal experiments and interventional study in ASD, Zilkha, N. et al. (35) found that a high-fat and imbalanced diet exacerbates cognitive rigidity and social deficiency in the BTBR mouse model of autism. A randomized controlled trial showed that diet modification based on the *Chanyi* approach in 1 month had positive effects on improving mental flexibility, response inhibition, and planning in children with ASD (36). However, it is controversial on the correlation between dietary quality and specific components of executive functions in normal population. Some studies (37–42) suggested that dietary quality was associated with inhibitory control, while the other studies (22, 43–46) showed that there was no association between dietary quality and inhibitory control. On the other hand, some studies (47–49) suggested that dietary quality was associated with working memory, while the other studies (47, 50–54) showed that there was no association between dietary quality and working memory. We speculated that compared to the TD children, the organizable ability and working memory in children with ASD may be more susceptible to dietary quality.

A moderate supply of nutrients could support the morphological development, neurochemistry, and neurophysiology of the human brain (17). First, the transfer of energy from foods to neurons might be fundamental to the control of brain function. Processes related to the management of energy in neurons can affect synaptic plasticity and have the potential to affect cognitive function (18). Second, some dietary components have been identified as having positive effects on cognitive ability (18, 55), such as minerals (e.g., Zn) and certain vitamins (e.g., B vitamins) are cofactors for enzymes that synthesize neurotransmitters, which could influence the development of cognition throughout childhood (56, 57); Dietary lipids (e.g., Omega-3 polyunsaturated fatty acids) can affect myelin sheath integrity and nerve cell membranes, so as to impact the brain function (18). Third, growing evidence indicated the importance of dietary components in influencing non-genetic events (e.g., DNA methylation, transcriptional activation, translational control, and posttranslational modifications), which cause a potentially heritable phenotypic change. In addition, there was evidence that dietary quality can influence the gut microbiome (58), and changes in the gut microbiome may impact executive functions through the “gut-brain axis” (19). Therefore, poor dietary quality may lead to changes in the brain structure and function, particularly in the brain regions associated with cognitive function (i.e., frontal cortical regions) involved, through synapse formation, in neurogenesis, myelination. The dimension of working memory refers to the ability to hold information in their mind and manipulate this information to achieve task completion, and it has been related to the frontal regions including the bilateral superior and middle frontal gyri, bilateral frontal polar regions, and precuneus gyrus (59). The dimension of organization represents the individual's organization within their environment and extends to the state of their work, living, and storage spaces (60), which involved the brain region including the dorsolateral prefrontal cortex, the anterior and posterior cingulate areas, and the parietal cortex (59, 61). The brain function of working memory and organization involved might be sensitive to the insufficient specific nutrients or energy which caused by insufficient dietary intake (i.e., cereals, animal foods, vegetable and fruit, dairy product). On the other hand, excessive dietary intake refers to the excess of foods that are emphasized as “less to eat” and “appropriate amount” in the Dietary Guidelines (i.e., cereals, animal foods, empty energy food and condiments), which might affect the function of brain regions involved by organizable ability through energy metabolism and other pathways. But the direction of the effects could also be reversed. Another possibility is that the impairment of executive function results in poor dietary quality. For example, studies have shown that inhibitory control may play a role in inhibiting dietary-related ideas or appetitive behaviors, and working memory may help children with better dietary quality by maintaining and updating dietary-related strategy (62). In future, more studies are needed to explore the underlying mechanisms involved.

Furthermore, we have compared the difference on the dietary quality and executive function between children with ASD and TD children to understand the association among them well. We found that compared to TD children, children with ASD had more problems of dietary quality including overall insufficient and unbalanced dietary intake. Further analysis showed that children with ASD had more insufficient dietary intake in the fruit, dairy and dietary variety categories than TD children. A few studies showed that there was no difference in dietary intake between children with ASD and TD children (63–65), however, the majority of studies showed similar results to ours (66–68). Insufficient and unbalanced dietary intake problems may be caused by the following factors, First, children with ASD often exhibit repetitive and stereotypical behaviors, and other uncontrolled behaviors at mealtimes (69, 70). These behaviors put them at higher risk for eating problems such as food rejection, high frequency of single food intake, pica (71–73). Second, dietary quality generally improved with the increase of income level and education level (74–77). However, children with ASD in the current study had lower family income and maternal education levels compared to TD children. Third, children with ASD are more likely to present with food allergies include especially milk/dairy, nuts, and fruits (78–81), which may lead to insufficient and unbalanced dietary intake. It is well known that dietary quality is a vital determinant of physical growth and mental development, poor dietary quality can contribute to non-communicable diseases (82), lead to worse mental health (83), and impair brain integrity and functionality (84), therefore, more attention should pay to the dietary management of children with ASD. Besides, we found that compared to TD children, children with ASD performed significantly worse on all subscales of BRIEF and working memory test, but had intact performance in SCWT. Executive function has long been considered to play a role in the specific impairments of ASD, such as social impairment, restricted and repetitive behavior patterns. However, the characteristic of executive function in ASD remains unclear because different studies used different assessment tool (33). Most studies used BRIEF to assess manifestations of executive function in everyday life of children, and showed that children with ASD had the impairment of executive functions, which suggested that children with ASD might have specific difficulties in complex, unstructured everyday problem-solving situations. However, it should be considered that BRIEF was parents- or teachers- reported which was sensitive for rater bias (85). Conversely, experimental tests (e.g., working memory test and SCWT) were administered directly to children with more objective. Most studies indicated that working memory in children with ASD had impairment (86). However, the impairment of inhibitory control in autistic children without ID was less prominent compared to the impairment of working memory (87). Most of the children with ASD in the current study were without ID, and most of them were recruited from mainstream educational schools, these might be why we found that children with ASD had relatively normal inhibitory control ability but poor working memory.

The current study had several strengths. First, it was the first study using the DBI_16 scores to evaluate the dietary quality of Chinese children with ASD and explore the association of dietary quality with executive functions in children with ASD. Second, the executive function of children with ASD was evaluated by both objective and subjective measures (i.e., BRIEF, working memory subscale of WISC-IV, and SCWT) to comprehensively reflect children's reality-based executive functions. Third, the dietary quality was assessed by DBI_16 rather than single food intake, which can represent the overall dietary information of children with ASD. However, some limitations should be considered. First, the current study was a cross-sectional design, thus it was impossible to determine the causal nature of the association between dietary quality and executive function in children with ASD. Further longitudinal studies or interventional studies are needed to confirm the relationship. Second, ASD represents a heterogeneity group of disorders, and most of the children with ASD in the current study were without ID, so the results couldn't reflect the association in autistic children with ID directly. Third, the study did not explore the relationship between dietary quality and cognitive flexibility which was an important component of executive function, and more studies were needed. Fourth, the dietary assessment in the current study was subjective reported, which could not accurately calculate the specific amounts of macronutrients and determine the intrinsic nutrient levels. Furthermore, the self-reported investigation might be influenced by parents' recall bias and personal expectations. Therefore, more objective investigations are needed to comprehensively collect children's dietary intake information.

## Conclusion

Poor dietary quality was associated with the impairment of working memory and organizational capacity in children with ASD. Compared to TD children, children with ASD had poorer dietary quality and executive functions. This study emphasized the importance of dietary quality in executive functions among children with ASD, and attention should be paid to improving their dietary quality.

## Data availability statement

The raw data supporting the conclusions of this article will be made available by the authors, without undue reservation.

## Ethics statement

The studies involving human participants were reviewed and was approved by the Ethical Review Committee for Biomedical Research, Sun Yat-Sen University (2015-No.29). Written informed consent to participate in this study was provided by the participants' legal guardian/next of kin.

## Author contributions

JJ and XL: conceptualization, supervision, project administration, and funding acquisition. XW and XS: methodology, formal analysis, and writing-original draft preparation. XW: study design, manuscript revise, and data curation. XW, XS, YJ, XZ, MC, XG, SL, XO, and TG: data collection. XS, XW, LC, and XL: writing - review and editing. All authors read and revised the manuscript, and approved the final manuscript.

## Funding

This work was supported by the Key-Area Research and Development Program of Guangdong Province (2019B030335001), the National Natural Science Foundation of China (81872639), and Guangdong Basic and Applied Basic Research Foundation (2021A1515011757).

## Conflict of interest

The authors declare that the research was conducted in the absence of any commercial or financial relationships that could be construed as a potential conflict of interest.

## Publisher's note

All claims expressed in this article are solely those of the authors and do not necessarily represent those of their affiliated organizations, or those of the publisher, the editors and the reviewers. Any product that may be evaluated in this article, or claim that may be made by its manufacturer, is not guaranteed or endorsed by the publisher.

## References

[1] Lord C Elsabbagh M Baird G Veenstra-Vanderweele J. Autism spectrum disorder. Lancet. (2018) 392:508–20. 10.1016/S0140-6736(18)31129-2 30078460 PMC7398158

[2] Zeidan J Fombonne E Scorah J Ibrahim A Durkin MS Saxena S. Global prevalence of autism: a systematic review update. Autism Res. (2022) 15:778–90. 10.1002/aur.2696 35238171 PMC9310578

[3] Baxter AJ Brugha TS Erskine HE Scheurer RW Vos T Scott JG. The epidemiology and global burden of autism spectrum disorders. Psychol Med. (2015) 45:601–13. 10.1017/S003329171400172X 25108395

[4] Garon N Bryson SE Smith IM. Executive function in preschoolers: a review using an integrative framework. Psychol Bull. (2008) 134:31–60. 10.1037/0033-2909.134.1.31 18193994

[5] Hill EL. Executive dysfunction in autism. Trends Cogn Sci. (2004) 8:26–32. 10.1016/j.tics.2003.11.003 14697400

[6] Perone S Simmering VR Buss AT. A dynamical reconceptualization of executive-function development. Perspect Psychol Sci. (2021) 16:1198–208. 10.1177/1745691620966792 33593126 PMC8364921

[7] Leung RC Vogan VM Powell TL Anagnostou E Taylor MJ. The role of executive functions in social impairment in autism spectrum disorder. Child Neuropsychol. (2016) 22:336–44. 10.1080/09297049.2015.1005066 25731979

[8] Mostert-Kerckhoffs MA Staal WG Houben RH de Jonge MV. Stop and change: inhibition and flexibility skills are related to repetitive behavior in children and young adults with autism spectrum disorders. J Autism Dev Disord. (2015) 45:3148–58. 10.1007/s10803-015-2473-y 26043846 PMC4569655

[9] de Vries M Geurts H. Influence of autism traits and executive functioning on quality of life in children with an autism spectrum disorder. J Autism Dev Disord. (2015) 45:2734–43. 10.1007/s10803-015-2438-1 25835211 PMC4553152

[10] Gisbert Gustemps L Lugo Marin J Setien Ramos I Ibanez Jimenez P Romero Santo-Tomas O Jurado Luque MJ. Sleep disturbances in autism spectrum disorder without intellectual impairment: relationship with executive function and psychiatric symptoms. Sleep Med. (2021) 83:106–14. 10.1016/j.sleep.2021.04.022 33991890

[11] van der Niet AG Smith J Scherder EJA Oosterlaan J Hartman E Visscher C. Associations between daily physical activity and executive functioning in primary school-aged children. J Sci Med Sport. (2015) 18:673–7. 10.1016/j.jsams.2014.09.006 25262450

[12] Bauermeister S Bunce D. Poorer mental health is associated with cognitive deficits in old age. Neuropsychol Dev Cogn B Aging Neuropsychol Cogn. (2015) 22:95–105. 10.1080/13825585.2014.893554 24605782

[13] Evans EW Must A Anderson SE Curtin C Scampini R Maslin M. Dietary patterns and body mass index in children with autism and typically developing children. Res Autism Spectr Disord. (2012) 6:399–405. 10.1016/j.rasd.2011.06.014 22936951 PMC3427936

[14] Meguid NA Anwar M Bjorklund G Hashish A Chirumbolo S Hemimi M. Dietary adequacy of Egyptian children with autism spectrum disorder compared to healthy developing children. Metab Brain Dis. (2017) 32:607–15. 10.1007/s11011-016-9948-1 28074329

[15] Tsujiguchi H Miyagi S Nguyen TTT Hara A Ono Y Kambayashi Y. Relationship between autistic traits and nutrient intake among Japanese children and adolescents. Nutrients. (2020) 12:2258. 10.3390/nu12082258 32731611 PMC7468902

[16] Liu X Liu J Xiong X Yang T Hou N Liang X. Correlation between nutrition and symptoms: nutritional survey of children with autism spectrum disorder in Chongqing, China. Nutrients. (2016) 8:294. 10.3390/nu8050294 27187463 PMC4882707

[17] Mahmassani HA Switkowski KM Scott TM Johnson EJ Rifas-Shiman SL Oken E. Maternal diet quality during pregnancy and child cognition and behavior in a US cohort. Am J Clin Nutr. (2022) 115:128–41. 10.1093/ajcn/nqab325 34562095 PMC8755080

[18] Gómez-Pinilla F. Brain foods: the effects of nutrients on brain function. Nat Rev Neurosci. (2008) 9:568–78. 10.1038/nrn2421 18568016 PMC2805706

[19] Davidson GL Cooke AC Johnson CN Quinn JL. The gut microbiome as a driver of individual variation in cognition and functional behaviour. Philos Trans R Soc Lond B Biol Sci. (2018) 373:20170286. 10.1098/rstb.2017.0286 30104431 PMC6107574

[20] Smyth A Dehghan M O'Donnell M Anderson C Teo K Gao P. Healthy eating and reduced risk of cognitive decline: a cohort from 40 countries. Neurology. (2015) 84:2258–65. 10.1212/WNL.0000000000001638 25948720 PMC4456656

[21] Riggs NR Spruijt-Metz D Chou CP Pentz MA. Relationships between executive cognitive function and lifetime substance use and obesity-related behaviors in fourth grade youth. Child Neuropsychol. (2012) 18:1–11. 10.1080/09297049.2011.555759 21480013

[22] Guerrieri R Nederkoorn C Jansen A. The interaction between impulsivity and a varied food environment: its influence on food intake and overweight. Int J Obes (Lond). (2008) 32:708–14. 10.1038/sj.ijo.0803770 18059403

[23] Pentz MA Spruijt-Metz D Chou CP Riggs NR. High calorie, low nutrient food/beverage intake and video gaming in children as potential signals for addictive behavior. Int J Environ Res Public Health. (2011) 8:4406–24. 10.3390/ijerph8124406 22408581 PMC3290975

[24] Qian Y Wang YF. [Reliability and validity of behavior rating scale of executive function parent form for school age children in China]. Beijing Da Xue Xue Bao Yi Xue Ban. (2007) 39:277–83. 10.19723/j.issn.1671-167x.2007.03.015 17572784

[25] Stroop JR. Studies of interference in serial verbal reactions. J Exp Psychol. (1935) 18:643–62. 10.1037/h0054651

[26] Lyu Y-J Lai L-J Li D-L. Reliability and validity of the questionnaire of surveillance on students' constitution and health for primary school students in Guangzhou. Matern Child Health Care China. (2020) 35:1511–6. 10.19829/j.zgfybj.issn.1001-4411.2020.08.047

[27] He Y Fang Y Xia J. Update of the Chinese diet balance. index:DBI_16. Acta Nutrimenta Sinica. (2018) 40:526–30. 10.13325/j.cnki.acta.nutr.sin.2018.06.003

[28] National Health Commission of the People's Republic of China. Screening for Overweight And Obesity Among School-Age Children and Adolescents. (2018). Available online at: http://www.nhc.gov.cn/wjw/pqt/201803/a7962d1ac01647b9837110bfd2d69b26.shtml (accessed March 30, 2018).

[29] Guidelines for Data Processing and Analysis of the International Physical Activity Questionnaire (IPAQ)-Short Form. (2004). Available online at: http://www.ipaq.ki.se (accessed April 1, 2004).

[30] Zhang HC. Revision of the Chinese version of Wechsler Intelligence Scale for children, Fourth vision. Pychol Sci. (2009) 32:1177-1179. 10.16719/j.cnki.1671-6981.2009.05.026

[31] R Core Team. R: A Language and Environment for Statistical Computing. R Foundation for Statistical Computing, Vienna, Austria. (2019). Available online at: https://www.R-project.org/

[32] Mangum RW Miller JS Brown WS Nolty AAT Paul LK. Everyday executive function and self-awareness in agenesis of the corpus callosum. J Int Neuropsychol Soc. (2021) 27:1037–47. 10.1017/S1355617721000096 33749569 PMC8744483

[33] Demetriou EA Lampit A Quintana DS Naismith SL Song YJC Pye JE. Autism spectrum disorders: a meta-analysis of executive function. Mol Psychiatry. (2018) 23:1198–204. 10.1038/mp.2017.75 28439105 PMC5984099

[34] Roth RM Erdodi LA McCulloch LJ Isquith PK. Much ado about norming: the behavior rating inventory of executive function. Child Neuropsychol. (2015) 21:225–33. 10.1080/09297049.2014.897318 24650292

[35] Zilkha N Kuperman Y Kimchi T. High-fat diet exacerbates cognitive rigidity and social deficiency in the BTBR mouse model of autism. Neuroscience. (2017) 345:142–54. 10.1016/j.neuroscience.2016.01.070 26855190

[36] Chan AS Sze SL Han YM Cheung MC. A chan dietary intervention enhances executive functions and anterior cingulate activity in autism spectrum disorders: a randomized controlled trial. Evid Based Complement Alternat Med. (2012) 2012:262136. 10.1155/2012/262136 22666288 PMC3359807

[37] Khan NA Raine LB Drollette ES Scudder MR Kramer AF Hillman CH. Dietary fiber is positively associated with cognitive control among prepubertal children. J Nutr. (2015) 145:143–9. 10.3945/jn.114.198457 25527669 PMC4264019

[38] Levitan RD Rivera J Silveira PP Steiner M Gaudreau H Hamilton J. Gender differences in the association between stop-signal reaction times, body mass indices and/or spontaneous food intake in pre-school children: an early model of compromised inhibitory control and obesity. Int J Obes. (2015) 39:614–9. 10.1038/ijo.2014.207 25512364

[39] Nederkoorn C Dassen FC Franken L Resch C Houben K. Impulsivity and overeating in children in the absence and presence of hunger. Appetite. (2015) 93:57–61. 10.1016/j.appet.2015.03.032 25841646

[40] Ames SL Kisbu-Sakarya Y Reynolds KD Boyle S Cappelli C Cox MG. Inhibitory control effects in adolescent binge eating and consumption of sugar-sweetened beverages and snacks. Appetite. (2014) 81:180–92. 10.1016/j.appet.2014.06.013 24949566 PMC4127340

[41] Whyte AR Schafer G Williams CM. Cognitive effects following acute wild blueberry supplementation in 7- to 10-year-old children. Eur J Nutr. (2016) 55:2151–62. 10.1007/s00394-015-1029-4 26437830

[42] Dohle S Diel K Hofmann W. Executive functions and the self-regulation of eating behavior: a review. Appetite. (2018) 124:4–9. 10.1016/j.appet.2017.05.041 28551113

[43] Pieper JR Laugero KD. Preschool children with lower executive function may be more vulnerable to emotional-based eating in the absence of hunger. Appetite. (2013) 62:103–9. 10.1016/j.appet.2012.11.020 23211377

[44] Stautz K Pechey R Couturier DL Deary IJ Marteau TM. Do executive function and impulsivity predict adolescent health behaviour after accounting for intelligence? Findings from the ALSPAC Cohort. PLoS One. (2016) 11:e0160512. 10.1371/journal.pone.0160512 27479488 PMC4968814

[45] Chung YC Park CH Kwon HK Park YM Kim YS Doo JK. Improved cognitive performance following supplementation with a mixed-grain diet in high school students: a randomized controlled trial. Nutrition. (2012) 28:165–72. 10.1016/j.nut.2011.05.017 22208555

[46] Ames SL Wurpts IC Pike JR MacKinnon DP Reynolds KR Stacy AW. Self-regulation interventions to reduce consumption of sugar-sweetened beverages in adolescents. Appetite. (2016) 105:652–62. 10.1016/j.appet.2016.06.036 27374899 PMC5443114

[47] Sheppard KW Cheatham CL. Executive functions and the ω-6-to-ω-3 fatty acid ratio: a cross-sectional study. Am J Clin Nutr. (2017) 105:32–41. 10.3945/ajcn.116.141390 27852615 PMC5183732

[48] Zhang J Hebert JR Muldoon MF. Dietary fat intake is associated with psychosocial and cognitive functioning of school-aged children in the United States. J Nutr. (2005) 135:1967–73. 10.1093/jn/135.8.1967 16046724

[49] Taib MN Shariff ZM Wesnes KA Saad HA Sariman S. The effect of high lactose-isomaltulose on cognitive performance of young children. A double blind cross-over design study. Appetite. (2012) 58:81–7. 10.1016/j.appet.2011.09.004 21986189

[50] Sheppard KW Cheatham CL. Omega-6 to omega-3 fatty acid ratio and higher-order cognitive functions in 7- to 9-y-olds: a cross-sectional study. Am J Clin Nutr. (2013) 98:659–67. 10.3945/ajcn.113.058719 23824723

[51] Kirby A Woodward A Jackson S Wang Y Crawford MA. A double-blind, placebo-controlled study investigating the effects of omega-3 supplementation in children aged 8-10 years from a mainstream school population. Res Dev Disabil. (2010) 31:718–30. 10.1016/j.ridd.2010.01.014 20171055

[52] Brindal E Baird D Slater A Danthiir V Wilson C Bowen J. The effect of beverages varying in glycaemic load on postprandial glucose responses, appetite and cognition in 10-12-year-old school children. Br J Nutr. (2013) 110:529–37. 10.1017/S0007114512005296 23244339

[53] Nyaradi A Foster JK Hickling S Li J Ambrosini GL Jacques A. Prospective associations between dietary patterns and cognitive performance during adolescence. J Child Psychol Psychiatry. (2014) 55:1017–24. 10.1111/jcpp.12209 24673485

[54] Kennedy DO Jackson PA Elliott JM Scholey AB Robertson BC Greer J. Cognitive and mood effects of 8 weeks' supplementation with 400 mg or 1,000 mg of the omega-3 essential fatty acid docosahexaenoic acid (DHA) in healthy children aged 10-12 years. Nutr Neurosci. (2009) 12:48–56. 10.1179/147683009X388887 19356306

[55] Cohen JF Gorski MT Gruber SA Kurdziel LB Rimm EB. The effect of healthy dietary consumption on executive cognitive functioning in children and adolescents: a systematic review. Br J Nutr. (2016) 116:989–1000. 10.1017/S0007114516002877 27487986

[56] Huskisson E Maggini S Ruf M. The influence of micronutrients on cognitive function and performance. J Int Med Res. (2007) 35:1–19. 10.1177/147323000703500101 17408051

[57] Black MM. Micronutrient deficiencies and cognitive functioning. J Nutr. (2003) 133:3927s−31s. 10.1093/jn/133.11.3927S 14672291 PMC3140638

[58] Singh RK Chang HW Yan D Lee KM Ucmak D Wong K. Influence of diet on the gut microbiome and implications for human health. J Transl Med. (2017) 15:73. 10.1186/s12967-017-1175-y 28388917 PMC5385025

[59] Demetriou EA DeMayo MM Guastella AJ. Executive function in autism spectrum disorder: history, theoretical models, empirical findings, and potential as an endophenotype. Front Psychiatry. (2019) 10:753. 10.3389/fpsyt.2019.00753 31780959 PMC6859507

[60] Ciszewski S Francis K Mendella P Bissada H Tasca GA. Validity and reliability of the behavior rating inventory of executive function - adult version in a clinical sample with eating disorders. Eat Behav. (2014) 15:175–81. 10.1016/j.eatbeh.2014.01.004 24854800

[61] Szczepanski SM Knight RT. Insights into human behavior from lesions to the prefrontal cortex. Neuron. (2014) 83:1002–18. 10.1016/j.neuron.2014.08.011 25175878 PMC4156912

[62] Groppe K Elsner B. The influence of hot and cool executive function on the development of eating styles related to overweight in children. Appetite. (2015) 87:127–36. 10.1016/j.appet.2014.12.203 25528693

[63] Shearer TR Larson K Neuschwander J Gedney B. Minerals in the hair and nutrient intake of autistic children. J Autism Dev Disord. (1982) 12:25–34. 10.1007/BF01531671 6896512

[64] Raiten DJ Massaro T. Perspectives on the nutritional ecology of autistic children. J Autism Dev Disord. (1986) 16:133–43. 10.1007/BF01531725 3722115

[65] Lockner DW Crowe TK Skipper BJ. Dietary intake and parents' perception of mealtime behaviors in preschool-age children with autism spectrum disorder and in typically developing children. J Am Diet Assoc. (2008) 108:1360–3. 10.1016/j.jada.2008.05.003 18656577

[66] Bjorklund G Meguid NA Dadar M Pivina L Kaluzna-Czaplinska J Jozwik-Pruska J. Specialized diet therapies: exploration for improving behavior in Autism Spectrum Disorder (ASD). Curr Med Chem. (2020) 27:6771–86. 10.2174/0929867327666200217101908 32065085

[67] Kuschner ES Eisenberg IW Orionzi B Simmons WK Kenworthy L Martin A. A preliminary study of self-reported food selectivity in adolescents and young adults with autism spectrum disorder. Res Autism Spectr Disord. (2015) 15:53–9. 10.1016/j.rasd.2015.04.005 26309446 PMC4545503

[68] Attlee A Kassem H Hashim M Obaid RS. Physical status and feeding behavior of children with autism. Indian J Pediatr. (2015) 82:682–7. 10.1007/s12098-015-1696-4 25663296

[69] Råstam M Täljemark J Tajnia A Lundström S Gustafsson P Lichtenstein P. Eating problems and overlap with ADHD and autism spectrum disorders in a nationwide twin study of 9- and 12-year-old children. Sci World J. (2013) 2013:315429. 10.1155/2013/315429 23690743 PMC3654255

[70] Sharp WG Berry RC McCracken C Nuhu NN Marvel E Saulnier CA. Feeding problems and nutrient intake in children with autism spectrum disorders: a meta-analysis and comprehensive review of the literature. J Autism Dev Disord. (2013) 43:2159–73. 10.1007/s10803-013-1771-5 23371510

[71] Postorino V Sanges V Giovagnoli G Fatta LM De Peppo L Armando M. Clinical differences in children with autism spectrum disorder with and without food selectivity. Appetite. (2015) 92:126–32. 10.1016/j.appet.2015.05.016 25998237

[72] Hyman SL Stewart PA Schmidt B Cain U Lemcke N Foley JT. Nutrient intake from food in children with autism. Pediatrics. (2012) 130:S145–53. 10.1542/peds.2012-0900L 23118245 PMC4536585

[73] Mari-Bauset S Zazpe I Mari-Sanchis A Llopis-Gonzalez A Morales-Suarez-Varela M. Food selectivity in autism spectrum disorders: a systematic review. J Child Neurol. (2014) 29:1554–61. 10.1177/0883073813498821 24097852

[74] Livingstone KM Olstad DL Leech RM Ball K Meertens B Potter J. Socioeconomic inequities in diet quality and nutrient intakes among australian adults: findings from a nationally representative cross-sectional study. Nutrients. (2017) 9:1092. 10.3390/nu9101092 28976927 PMC5691709

[75] Hiza HA Casavale KO Guenther PM Davis CA. Diet quality of Americans differs by age, sex, race/ethnicity, income, and education level. J Acad Nutr Diet. (2013) 113:297–306. 10.1016/j.jand.2012.08.011 23168270

[76] Lallukka T Laaksonen M Rahkonen O Roos E Lahelma E. Multiple socio-economic circumstances and healthy food habits. Eur J Clin Nutr. (2007) 61:701–10. 10.1038/sj.ejcn.1602583 17180154

[77] Huot I Paradis G Receveur O Ledoux M. Correlates of diet quality in the Quebec population. Public Health Nutr. (2004) 7:1009–16. 10.1079/PHN2004637 15548338

[78] Gurney JG McPheeters ML Davis MM. Parental report of health conditions and health care use among children with and without autism: national survey of children's health. Arch Pediatr Adolesc Med. (2006) 160:825–30. 10.1001/archpedi.160.8.825 16894082

[79] Lyall K Van de Water J Ashwood P Hertz-Picciotto I. Asthma and allergies in children with autism spectrum disorders: results from the CHARGE study. Autism Res. (2015) 8:567–74. 10.1002/aur.1471 25722050 PMC6900397

[80] Peretti S Mariano M Mazzocchetti C Mazza M Pino MC Verrotti Di Pianella A. Diet: the keystone of autism spectrum disorder? Nutr Neurosci. (2019) 22:825–39. 10.1080/1028415X.2018.1464819 29669486

[81] Panossian C Lyons-Wall P Whitehouse A Oddy WH Lo J Scott J. Young adults with high autistic-like traits displayed lower food variety and diet quality in childhood. J Autism Dev Disord. (2021) 51:685–96. 10.1007/s10803-020-04567-4 32617793 PMC7835288

[82] Dalwood P Marshall S Burrows TL McIntosh A Collins CE. Diet quality indices and their associations with health-related outcomes in children and adolescents: an updated systematic review. Nutr J. (2020) 19:118. 10.1186/s12937-020-00632-x 33099309 PMC7585689

[83] O'Neil A Quirk SE Housden S Brennan SL Williams LJ Pasco JA. Relationship between diet and mental health in children and adolescents: a systematic review. Am J Public Health. (2014) 104:e31–42. 10.2105/AJPH.2014.302110 25208008 PMC4167107

[84] Muth AK Park SQ. The impact of dietary macronutrient intake on cognitive function and the brain. Clin Nutr. (2021) 40:3999–4010. 10.1016/j.clnu.2021.04.043 34139473

[85] Blijd-Hoogewys EM Bezemer ML van Geert PL. Executive functioning in children with ASD: an analysis of the BRIEF. J Autism Dev Disord. (2014) 44:3089–100. 10.1007/s10803-014-2176-9 24996868

[86] Wang Y Zhang YB Liu LL Cui JF Wang J Shum DH. A meta-analysis of working memory impairments in autism spectrum disorders. Neuropsychol Rev. (2017) 27:46–61. 10.1007/s11065-016-9336-y 28102493

[87] Lai CLE Lau Z Lui SSY Lok E Tam V Chan Q. Meta-analysis of neuropsychological measures of executive functioning in children and adolescents with high-functioning autism spectrum disorder. Autism Res. (2017) 10:911–39. 10.1002/aur.1723 27874266

