# Supplementary material for: Association between dietary quality and executive functions in school-aged children with autism spectrum disorder
Source: Front Nutr. 2022 Aug 4;9:940246. doi: 10.3389/fnut.2022.940246 (PMC9386179; doi:10.3389/fnut.2022.940246)
Supplement: Supplementary file 1 [file Data_Sheet_1.docx]

Supplementary Material

Table.S1 Overview of the studies about the association between dietary quality and executive functions.

| **Year** | **Author** | **Country** | **Participants** | **Sample size** | **Age** | **Executive functions measures** | **Dietary measures** | **Dietary relation to executive functions** |
| --- | --- | --- | --- | --- | --- | --- | --- | --- |
| **Randomized controlled trial** | | | | | | | | |
| 2012 | Chan | China | ASD | 24 | 7-17 years | D2 Test of Concentration Go/No-Go Task Children's Color Trails Test The Five-Point Test The Tower of California Test | Specific Diet Modification. Experimental group children were recommended to reduce their intake of some foods | Experimental group demonstrated significantly improved mental flexibility and inhibitory control after the diet modification |
| 2010 | Kirby | UK | TD | 235 | 8-9 years | Working Memory Test Battery for Children | Supplements given to children then cheek cell analysis of fatty acid conducted to verify consumption | No relation between supplementation of omega-3 fatty acids and performance on the Working Memory Test Battery for Children after 24 weeks |
| 2016 | Whyte | UK | TD | 21 | 7-10 years | Go/No-Go Task Flanker Task | Lab-Based Food Task | Positive relation between consumption of a wild blueberry powder drink and performance on Flanker Task No relation between consumption of a wild blueberry powder drink and performance on a Go/No-Go Task |
| 2016 | Ames | USA | TD | 168 | 14-17 years | Inhibition Survey for recruitment; Go/No Go Task for intervention; Implementation Intentions for intervention; Self-Ordered Point Task for working memory moderation analyses | Food Frequency Questionnaire Lab-Based Food Task | No relation between sugar sweetened beverage (SSB) and Go/No-Go Task training No relation between SSB related Implementation Intentions and sugar consumed, or type of beverages consumed in the lab. Negative effect of completing SSB related Implementation Intentions as compared with control condition on calories consumed and total amount of sugar consumed. Negative effect of completing SSB related Implementation Intentions and participating in SSB related Go/No-Go Task as compared with control condition on choosing unhealthy drinks and grams of sugar consumed No effect of completing SSB related Go/No-Go Task on number of calories consumed, number of calories consumed from beverages, grams of sugar consumed, or grams of sugar from drinks consumed. No moderation or main effect of working memory on any of the analyse |
| 2011 | Chung | Korea | TD | 28 | Tenth, Eleventh Grade Years | Computerized Neuro-psychological Test (CNT)-Stroop Task | Meals provided to participants and complicance checks conducted | No relation between diet type and (mixed grain or control group) performance on Stroop Task after 9 weeks |
| 2012 | Mohd Taib | Malaysia | TD | 30 | Kindergarten year | Cognitive Drug Research Battery (CDR)-Computerized Spatial Working Memory Task and Numeric Working Memory Task | Lab-Based Food Task | Positive relation between consumption of glucose fortified drink and spatial working memory. Positive relation between isomaltulose drink and numeric working memory. Better numeric working memory in children who received isomaltulose drink as compared with reformulated lactose drink or glucose drink |
| 2013 | Brindal | Australia | TD | 40 | 10-12 years | Attention Switching Task | Lab-Based Food Task | No relation between test drink consumed and Attention Switching Task performance |
| 2014 | Nyaradi | Australia | TD | 589 | 14 years | Groton Maze Learning Task | Food Frequency Questionnaire "Healthy" and "Western" dietary patterns were identified by factor analysis. | No relation between “healthy” diet (e.g., high in fruits, vegetables, whole grains, legumes, fish, versus “Western”) at age 14 and errors in performance on Groton Maze Learning Task at age 17 Positive relation between “Western” diet (e.g., high in take-out, red meat, soft drinks, fried/refined foods, versus “Healthy”) at age 14 and errors in performance on Groton Maze Learning Task, Delayed Recall at age 17 |
| 2009 | Kennedy | UK | TD | 90 | 10-12 years | CDR Battery-Computerized Numeric Working Memory Task | Diary card to conduct compliance with intervention | No relation between any level of DHA supplementation and performance on Numeric Working Memory Task after 56 days of supplementation |
| **Longitudinal** | | | | | | | | |
| 2010 | Riggs | USA | TD | 184 | Fourth grade year | Behavior Rating Inventory of Executive Function (BRIEF-SR) | Food Frequency Questionnaire Calculated the intake of Snack food, Vegetable and Fruit | No relation between BRIEF-SR scores and snack food consumption Positive relation between BRIEF-SR scores and fruit/vegetable consumption |
| 2015 | Tate | USA | TD | 1005 | Fourth grade year | Behavior Rating Inventory of Executive Function (BRIEF-SR) | Food Frequency Questionnaire Calculated the intake of Snack food | No relation between BRIEF-SR scores and “high-calorie low nutrient” snack food consumption |
| **Cohort** | | | | | | | | |
| 2016 | Stautz | UK | TD | 6069 | 7-13 years | Stop Signal Task | Food Diary Calculated the average amount of fruit and vegetables, in grams, consumed per day over the three day period | No relation between Stop-Signal Task performance and fruit and vegetable consumption |
| **Cross-sectional** | | | | | | | | |
| 2012 | Riggs | USA | TD | 1587 | Fourth grade year | Behavior Rating Inventory of Executive Function (BRIEF-SR) | Food Frequency Questionnaire Calculated the intake of Snack food, Vegetable and Fruit | Negative relation between BRIEF-SR scores and snack food consumption Positive relation between BRIEF-SR scores and fruit/vegetable consumption |
| 2010 | Riggs | USA | TD | 107 | Fourth grade year | Behavior Rating Inventory of Executive Function (BRIEF-SR) | Food Frequency Questionnaire Calculated the intake of Snack food, Vegetable and Fruit | Negative relation between BRIEF-SR scores and snack food consumption No relation between BRIEF-SR scores and fruit or vegetable intake |
| 2015 | Khan | USA | TD | 65 | 7-9 years | Flanker Task | Three-day food records were used to conduct nutrient-level analyses and to calculate diet quality (Healthy Eating Index-2005) scores | Positive relation between Flanker task performance and dietary fiber intake, overall diet quality Negative relation between Flanker task performance and fat, cholesterol intake |
| 2015 | Levitan | Canadian | TD | 193 | 48 months | Stop-Signal Task | Lab-Based Food Task Calculated the intake of fat, carbohydrates and protein | Negative relation between Stop-Signal Task performance and intake of carbohydrates, sugars No relation between Stop-Signal Task performance and total caloric intake, intake of protein, fats |
| 2015 | Nederkoorn | Netherlands | TD | 88 | 7-9 years | Stop-Signal Task | Lab-Based Food Task Measured caloric intake | Negative relation between Stop-Signal Task performance and intake of candy, chips |
| 2014 | Ames | USA | TD | 198 | 14-17 years | Go/No-Go Task | Food Frequency Questionnaire Calculated average intake | Negative relation between Go/No-Go Task performance and sweet, salty/fatty snack consumption(males) No relation between Go/No-Go Task performance and sweet, salty/fatty snack consumption(females) No relation between Go/No-Go Task performance and sugar sweetened beverage consumption(males or females) |
| 2008 | Guerrieri | Netherlands | TD | 78 | 8-10 years | Stop-Signal Task | Lab-Based Food Task Calculated total caloric intake | No relation between Stop-Signal Task performance and caloric intake |
| 2013 | Pieper | USA | TD | 29 | 3-6 years | Flanker Task | Lab-Based Food Task Measured caloric intake | No relation between calories consumed (total, sweet or savory) and performance on the Flanker Task |
| 2017 | Sheppard | USA | TD | 41 | 7-9 years | CANTAB-Computerized Spatial Working Memory Task | 24-Hour Dietary Recall Blood samples were collected for fatty acids identification and quantification | No relation between n-6: n-3 fatty acid ratio or mean n-3 fatty acid intake and Spatial Working Memory Task performance |
| 2005 | Zhang | USA | TD | 3666 | 6-16 years | Digit Span subtests-Wechsler Intelligence Scale for Children Revised (WISC-R) | 24-Hour Dietary Recall  Estimated dietary intakes of total fat, SFA, monounsaturated fatty, PUFAs, and cholesterol | Positive relation between Digit Span performance and polyunsaturated fat consumption Negative relation between Digit Span performance and cholesterol consumption No relation between Digit Span performance and other dietary variables |
| 2013 | Sheppard | USA | TD | 69 | 7-9 years | Cambridge Neuropsychological Test Assessment Battery(CANTAB)-Spatial Span Task (computerized digit span task); Spatial Working Memory Task | 24-Hour Dietary Recall Calculated total n-6 fatty acids, total n-3 fatty acids, and kilocalorie intake | No relation between consumption of fatty acids and Spatial Span Task performance Trending positive relation between n:6-n:3 fatty acid ratio and performance on the Spatial Working Memory Task No relation between other consumption variables and Spatial Working Memory Task |

Table.S2 Components of DBI_16(1)

| **Components** | **Score** | **Subgroup** | **Score** | **Intake range by energy levels based on the *Chinese Dietary Guidelines* (2016)** | | | | | | | | | | |
| --- | --- | --- | --- | --- | --- | --- | --- | --- | --- | --- | --- | --- | --- | --- |
|  |  |  |  | **4180kJ** | **5020kJ** | **5860kJ** | **6700kJ** | **7550kJ** | **8350kJ** | **9200kJ** | **10050kJ** | **10900kJ** | **11700kJ** | **12550kJ** |
|  |  |  |  | **1000kcal** | **1200kcal** | **1400kcal** | **1600kcal** | **1800kcal** | **2000kcal** | **2200kcal** | **2400kcal** | **2600kcal** | **2800kcal** | **3000kcal** |
| C1- Cereal* | (-12)-12 | Cereal | (-12)-12^#^ | 0g=-12 | <15g=-12 | 0g=-12 | <10g=-12 | <35g=-12 | <5g=-12 | <30g=-12 | 0g=-12 | 0g=-12 | <75g=-12 | <100g=-12 |
|  |  |  |  | 75-95g=0 | 90-110g=0 | 125-175g=0 | 175-225g=0 | 200-250g=0 | 225-275g=0 | 250-300g=0 | 275-325g=0 | 275-325g=0 | 350-400g=0 | 375-425g=0 |
|  |  |  |  | >170g=12 | >185g=12 | >250g=12 | >390g=12 | >415g=12 | >495g=12 | >520g=12 | >600g=12 | >600g=12 | >675g=12 | >700g=12 |
| C2- Vegetable and fruit | (-12)-0 | Vegetable | (-6)-0 | ≥ 200g=0 | ≥ 250g=0 | ≥ 300g=0 | | ≥ 400g=0 | ≥ 450g=0 | | ≥ 500g=0 | | | ≥ 600g=0 |
|  |  |  |  | 160-199g=-1 | 200-249g=-1 | 240-299g=-1 | | 320-399g=-1 | 360-449g=-1 | | 400-499g=-1 | | | 480-599g=-1 |
|  |  |  |  | score decreased 1 with intake amount decreased 40g | score decreased 1 with intake amount decreased 50g | score decreased 1 with intake amount decreased 60g | | score decreased 1 with intake amount decreased 80g | score decreased 1 with intake amount decreased 90g | | score decreased 1 with intake amount decreased 100g | | | score decreased 1 with intake amount decreased 120g |
|  |  |  |  | 0g=-6 | 0g=-6 | 0g=-6 | | 0g=-6 | 0g=-6 | | 0g=-6 | | | 0g=-6 |
|  |  | Fruit | (-6)-0 | ≥ 150g=0; 120-149g=-1 | | | ≥ 200g=0; 160-199g=-1 | | ≥ 300g=0; 240-299g=-1 | | ≥ 350g=0; 280-349g=-1 | | ≥ 400g=0; 320-399g=-1 | |
|  |  |  |  | score decreased 1 with intake amount decreased 30g | | | score decreased 1 with intake amount decreased 40g | | score decreased 1 with intake amount decreased 60g | | score decreased 1 with intake amount decreased 70g | | score decreased 1 with intake amount decreased 80g | |
|  |  |  |  | 0g=-6 | | | 0g=-6 | | 0g=-6 | | 0g=-6 | | 0g=-6 | |

| (Continued) | | | | | | | | | | | | | | |
| --- | --- | --- | --- | --- | --- | --- | --- | --- | --- | --- | --- | --- | --- | --- |
| **Components** | **Score** | **Subgroup** | **Score** | **Intake range by energy levels based on the *Chinese Dietary Guidelines* (2016)** | | | | | | | | | | |
|  |  |  |  | **4180kJ** | **5020kJ** | **5860kJ** | **6700kJ** | **7550kJ** | **8350kJ** | **9200kJ** | **10050kJ** | **10900kJ** | **11700kJ** | **12550kJ** |
|  |  |  |  | **1000kcal** | **1200kcal** | **1400kcal** | **1600kcal** | **1800kcal** | **2000kcal** | **2200kcal** | **2400kcal** | **2600kcal** | **2800kcal** | **3000kcal** |
| C3-Milk and dairy products Soybean and soybean products | (-12)-0 | Dairy | (-6)-0 | ≥ 500g=0, | | ≥ 350g=0, | ≥ 300g=0, score decreased 1 with intake amount decreased 60g | | | | | | | |
|  |  |  |  | score decreased 1 with intake amount decreased 100g | | score decreased 1 with intake amount decreased 70g | 0g=-6 | | | | | | | |
|  |  |  |  | 0g=-6 | | 0g=-6 |  | | | | | | | |
|  |  | Soybean | (-6)-0 | ≥ 5g=0 | ≥ 15g=0 | | | | | ≥ 25g=0 | | | | |
|  |  |  |  | score decreased 1 with intake amount decreased 1g | score decreased 1 with intake amount decreased 3g | | | | | score decreased 1 with intake amount decreased 5g | | | | |
|  |  |  |  | 0g=-6 | 0g=-6 | | | | | 0g=-6 | | | | |

| (Continued) | | | | | | | | | | | | | | |
| --- | --- | --- | --- | --- | --- | --- | --- | --- | --- | --- | --- | --- | --- | --- |
| **Components** | **Score** | **Subgroup** | **Score** | **Intake range by energy levels based on the *Chinese Dietary Guidelines* (2016)** | | | | | | | | | | |
|  |  |  |  | **4180kJ** | **5020kJ** | **5860kJ** | **6700kJ** | **7550kJ** | **8350kJ** | **9200kJ** | **10050kJ** | **10900kJ** | **11700kJ** | **12550kJ** |
|  |  |  |  | **1000kcal** | **1200kcal** | **1400kcal** | **1600kcal** | **1800kcal** | **2000kcal** | **2200kcal** | **2400kcal** | **2600kcal** | **2800kcal** | **3000kcal** |
| C4- Animal food | (-12)-8 | Red meat and products, Poultry and game | (-4)-4 | 0g=-3 | 0g=-4 | 0g=-4 | | 0g=-4 | | 0g=-4 | | | 0g=-4 | |
|  |  |  |  | 1-5g=-2 | 1-5g=-3 | 1-10g=-3 | | 1-15g=-3 | | 1-20g=-3 | | | 1-25g=-3 | |
|  |  |  |  | 6-10g=-1 | 6-10g=-2 | 11-20g=-2 | | 16-30g=-2 | | 21-40g=-2 | | | 26-50g=-2 | |
|  |  |  |  | 11-20g=0 | 11-15g=-1 | 21-30g=-1 | | 31-45g=-1 | | 41-60g=-3 | | | 51-75g=-1 | |
|  |  |  |  | 21-25g=1 | 16-35g=0 | 31-50g=0 | | 46-55g=0 | | 61-90g=0 | | | 76-125g=0 | |
|  |  |  |  | 26-30g=2 | 36-40g=1 | 51-60g=1 | | 56-70g=1 | | 91-110g=1 | | | 126-150g=1 | |
|  |  |  |  | 31-35g=3 | 41-45g=2 | 61-70g=2 | | 71-85g=2 | | 111-130g=2 | | | 151-175g=2 | |
|  |  |  |  | >35g =4 | 46-50g=3 | 71-80g=-3 | | 85-100g=3 | | 131-150g=3 | | | 176-200g=3 | |
|  |  |  |  |  | >50g =4 | >80g =4 | | >100g =4 | | >150g=4 | | | >200g=4 | |
|  |  | Fish and shrimp | (-4)-0 | 0g=-4 | <5g=-4 | <10g=-4 | | <10g=-4 | | 0g=-4 | | | <25g=-4 | <50g=-4 |
|  |  |  |  | 1-4g=-3 | 5-9g=-3 | 10-19g=-3 | | 10-19g=-3 | | 1-24g=-3 | | | 25-49g=-3 | 50-74g=-3 |
|  |  |  |  | 5-9g=-2 | 10-14g=-2 | 20-29g=-2 | | 20-29g=-2 | | 25-49g=-2 | | | 50-74g=-2 | 75-99g=-2 |
|  |  |  |  | 10-14g=-1 | 15-19g=-1 | 30-39g=-1 | | 30-39g=-1 | | 50~74g=-1 | | | 75-99g=-1 | 100-124g=-1 |
|  |  |  |  | ≥ 15g=0 | ≥ 20g=0 | ≥ 40g=0 | | ≥ 40g=0 | | ≥ 75g=0 | | | ≥ 100g=0 | ≥ 125g=0 |
|  |  | Egg | (-4)-4 | 0g=-4 ; | <5g=-4;6-10g=-3;11-15g= | | 0g=-4;1-10g=-3;11-20g=- | | 0g=-4;1-15g=-3;16-30g=-2;31-45g=-1;46-55g=0;56-70g=1;71-85g=2;85-100g= | | | | | |
|  |  |  |  | 1-5g=-3; | -2;16-20g=-1;21-30g=0;3 | | 2;21-30g=-1;31-50g=0;51 | | 3;>100g =4 | | | | | |
|  |  |  |  | 6-10g=-2; | 1-35g=1;36-40g=2;41-45g | | -60g=1;61-70g=2;71-80g | |  | | | | | |
|  |  |  |  | 11-15g=-1; | =3;>45g =4 | | =-3;>80g =4 | |  | | | | | |
|  |  |  |  | 16-25g=0; |  | |  | |  | | | | | |
|  |  |  |  | 26-30g=1; |  | |  | |  | | | | | |
|  |  |  |  | 31-35g=2; |  | |  | |  | | | | | |
|  |  |  |  | 36-40g=3; |  | |  | |  | | | | | |
|  |  |  |  | >40g =4 |  | |  | |  | | | | | |

| (Continued) | | | | | | | | | | | | | | |  |
| --- | --- | --- | --- | --- | --- | --- | --- | --- | --- | --- | --- | --- | --- | --- | --- |
| **Components** | **Score** | **Subgroup** | **Score** | **Intake range by energy levels based on the *Chinese Dietary Guidelines* (2016)** | | | | | | | | | | |  |
|  |  |  |  | **4180kJ** | **5020kJ** | **5860kJ** | **6700kJ** | **7550kJ** | **8350kJ** | **9200kJ** | **10050kJ** | **10900kJ** | **11700kJ** | **12550kJ** |  |
|  |  |  |  | **1000kcal** | **1200kcal** | **1400kcal** | **1600kcal** | **1800kcal** | **2000kcal** | **2200kcal** | **2400kcal** | **2600kcal** | **2800kcal** | **3000kcal** |  |
| C5-Empty energy food | 0-12 | Cooking oil | 0-6^##^ | ≤ 20g=0 | ≤ 25g=0 | | | | | | ≤ 30g=0 | | | ≤ 35g=0 |  |
|  |  |  |  | 21-25g=1 | 26-30g=1 | | | | | | 31-35g=1 | | | 36-40g=1 |  |
|  |  |  |  | >45g=6 | >50g=6 | | | | | | >55g=6 | | | >60g=6 |  |
|  |  | Alcoholic beverage | 0-6 | Male：≤ 25g=0 26-40g=1 26-100g score increased 1 with intake amount increased 15g >100g=6(25g alcohol=750ml beer or 250ml wine or 75g liquor (38°) or 50g liquor (> 38°)) | | | | | | | | | | |  |
|  |  |  |  | Female:≤15g=0 16g=1 16-25g=1 score increased 1 with intake amount increased 10g >65g=6(15g alcohol=450ml beer or 150ml wine or 50g liquor(38°) or 30g liquor(> 38°)) | | | | | | | | | | |  |
| C6-Condiments | 0-12 | Addible sugar | 0-6 | ≤ 25g=0 26g=1 score increased 1 with intake amount increased 5g >50g=6 | | | | | | | | | | |  |
|  |  |  |  |  |  |  |  |  |  |  |  |  |  |  |  |
| C7- Diet variety | (-12)-0 | Diet variety | (-12)-0 | ≥ 12 kinds of food (soybean is 5g)=0, score decreased 1 with decreased 1 kinds of food | | | | | | | | | | |  |
|  |  |  |  |  |  |  |  |  |  |  |  |  |  |  |  |
| C8- Drinking water | (-12)-0 | Drinking water | (-12)-0 | ≥ 1200ml=0 score decreased 1 with intake amount decreased 100ml, <100ml=-12 | | | | | | | | | | |  |
|  |  |  |  |  |  |  |  |  |  |  |  |  |  |  |  |
| *Cereal include rice, wheat, dried legumes (exclude soybean) and tubers. Intake amount means fresh amount. Sweat potato: intake amount divided by 3; potato: intake amount divided by 4; yam and yambean: divided by 6; score increased (decreased) 2 with 15g intake increased (decreased) when the energy intake level is 1000 and 1200; score increased (decreased) 2 with 25g intake increased (decreased) when the energy intake level is 1400 kcal; 1 with 15g intake for 1600 and 1800 kcal; 1 with 20g for 2000 and 2200 kcal; and 1 with 25g for the energy intake level more than 2400 kcal；  ^#^ score increased(decreased) 2 with 50g intake increased(decreased) from 0 to maximal (minimal) score；  ^##^ score increased 1 with intake amount increased 5g from score 1 to 6. | | | | | | | | | | | | | | | |

**Table.S3** **Comparison of DBI_16 indicator among children with ASD and TD children**

| DBI_16 indicator | ASD  N (%) | TD  N (%) | *P* value |
| --- | --- | --- | --- |
|  | N=106 | N=207 |  |
| HBS |  |  | 0.24 |
| No problem | 0 (0.0) | 2 (1.0) |  |
| Almost no problem | 73 (68.9) | 160 (77.3) |  |
| Low level | 29 (27.4) | 40 (19.3) |  |
| Moderate level and High level | 4 (3.8) | 5 (2.4) |  |
| LBS |  |  | **0.01** |
| No problem and almost no problem | 6 (5.7) | 15 (7.2) |  |
| Low level | 55 (51.9) | 141 (68.1) |  |
| Moderate level | 40 (37.7) | 48 (23.2) |  |
| High level | 5 (4.7) | 3 (1.4) |  |
| DQD |  |  | **0.01** |
| No problem and almost no problem | 3 (2.8) | 12 (5.8) |  |
| Low level | 64 (60.4) | 151 (72.9) |  |
| Moderate level and High level | 39 (36.8) | 44 (21.3) |  |
| **Abbreviation:** ASD, autism spectrum disorder; TD, typically developing; DBI_16, The Chinese Dietary Balance Index_16; LBS, Lower Bound Score; HBS, Higher Bound Score; DQD, Diet Quality Distance. | | | |

**Table.S4** **Comparison of** **DBI_16 components among children with ASD and TD children**

| DBI_16 components | ASD  Mean (SD) | TD  Mean (SD) | *P* value |
| --- | --- | --- | --- |
|  | N=106 | N=207 |  |
| Cereals | -1.2 (7.0) | 0.8 (6.0) | 0.64 |
| Vegetable | -4.0 (1.4) | -4.1 (1.1) | 0.63 |
| Fruit | -3.8 (1.4) | -3.4 (1.5) | **0.01** |
| Dairy | -2.3 (1.8) | -1.9 (1.6) | **0.02** |
| Soybean | -2.9 (2.3) | -2.6 (2.3) | 0.23 |
| Meat | 2.0 (2.4) | 2.2 (2.1) | 0.36 |
| Fish and shrimp | -2.4 (1.3) | -2.5 (1.2) | 0.67 |
| Egg | -0.2 (1.5) | -0.2 (1.7) | 0.86 |
| Cooking oil | 1.0 (0.5) | 0.9 (0.6) | 0.50 |
| Alcoholic beverage | 0 | 0 | - |
| Addible sugar | 2.4 (2.8) | 2.0 (2.6) | 0.18 |
| Salt | 0.9 (0.3) | 0.9 (0.4) | 0.13 |
| Variety | -4.1 (1.9) | -3.7 (1.9) | **0.047** |
| Water | -4.7 (3.3) | -4.7 (3.0) | 0.87 |

**Abbreviation:** ASD, autism spectrum disorder; TD, typically developing; SD, standard deviation; DBI_16, The Chinese Dietary Balance Index_16.

Reference

1. He Y, Fang Y, Xia J. Update of the Chinese Diet Balance Index:DBI_16. Acta Nutrimenta Sinica. 2018;40(6):526-30.
